# Announcement: Symposium on the Bioniorganic Chemistry of Nitrogen Oxides

**DOI:** 10.1155/MBD.1996.49

**Published:** 1996


					SYMPOSIUM
CHEMISTRY
ON
OF
THE BIONIORGANIC
NITROGEN OXIDES
Tuesday-Thursday, March 26-28
ACS National Meeting, New Orleans, Spring, 1996
Session I" Overview of the Chemistry and Biochemistry of Nitrogen Oxides
Prof. L. Ignarro, UCLA: The Biology of Nitric Oxide
Prof. J. Enemark, Arizona: Transition Metal Complexes of Nitrogen Oxides
Prof. W. Koppenol, ETH Zurich: Fundamental Chemistry of Nitrogen Oxides
Prof. S. Bohle, Wyoming: Synthesis and Characterization of Higher Nitrogen Oxides
Prof. J. Lipscomb, Minnesota: Nitric Oxide as a Probe of Metalloprotein Structure and Function
Session I1: The Chemistry and Biology of Nitrogen Oxide Reduction
Prof. B. Averill, Amsterdam: The Mechanisms of Cu and Fe Nitrite Reductases
Prof. P. Kroneck, Konstanz: Nitrous Oxide Reductase
Prof. E. Adman, Univ. Washington Structure and Function of Copper Nitrite Reductase
Prof. I. Moura, Lisbon: Hexaheme Nitrite Reductatses
Prof. W. Tolman, Minnesota: Synthetic Modeling of Copper Protein-Nitrogen Oxide Interactions
Session II1: NO as a Biological Signalling Agent.
The Chemistry of NO. Synthesis and Action
Prof. Dennis Stuehr, Cleveland Clinic: The Biosynthesis of NO by Nitric Oxide Synthase
Prof. J. Fukuto, UCLA: Modelling the Biosynthesis of NO
Prof. F.A. Walker, Arizona: A Novel NO Liberating Protein from a Bloodsucking Insect
Prof. J. Burstyn, Wisconsin: NO Activation of Soluble Guanylyl Cyclase
Dr. L. Keefer, NIH: NO-Releasing Complexes as Pharmaceutical Agents
Session IV: Fundamental Chemistry of Nitrogen Oxides and Their Complexes
Prof. R. Scheidt, Notre Dame: Iron Porphyrin NOx Chemistry
Prof. P. Legzdins, British Columbia: Reactivity of Metal Nitrosyl Complexes
Prof. T. Meyer, North Carolina: Mechanistic Studies of NOx Reduction by Iron Porphyrins
Prof. G. Richter-Addo, U. Oklahoma: Metalloporphyrin Complexes of NO and NO-donors
Prof. P. Ford, U. C. S. B.: Photochemistry of Iron and Copper Nitrosyls, Biological NO Sensing with
Model Complexes
Prof. Dean Wilcox, Dartmouth: Detection of NO in Biological Systems
There will be one general session and a designated section of the Inorganic poster session for
contributed papers associated with this symposium. Contributed abstracts should be sent on ACS
abstract forms to Prof. William Tolman at the University of Minnesota no later than December 1, 1995.
For more information, please contact
Judith N. Burstyn
Department of Chemistry
University of Wisconsin
1101 University Ave.
Madison, Wl 53706
Phone: (608)262-0328
FAX: (608)262-6143
E-MAIL: burstyn@chem.wisc.edu
This information is also posted on the ACS Division of Inorganic Chemistry WWW site
at:http://infoeagle.bc.edu/chemistry/Inorganic/Inorganic.html and the ACS DIC Bioinorganic
Subdivision site at: http://sbchml.sunysb.edu/koch/biic.html
49

				

